# Preparation, Structural Characterisation, and Bioactivities of Fructans: A Review

**DOI:** 10.3390/molecules28041613

**Published:** 2023-02-07

**Authors:** Min Wang, Kit-Leong Cheong

**Affiliations:** 1 Guangdong Provincial Key Laboratory of Aquatic Product Processing and Safety, Guangdong Province Engineering Laboratory for Marine Biological Products, Guangdong Provincial Engineering Technology Research Center of Seafood, Guangdong Provincial Science and Technology Innovation Center for Subtropical Fruit and Vegetable Processing, College of Food Science and Technology, Guangdong Ocean University, Zhanjiang 524088, China; 2 Postgraduate College, Guangdong Ocean University, Zhanjiang 524088, China

**Keywords:** fructan, polysaccharide, inulin, bioactivity, analysis

## Abstract

Polysaccharides are important components of higher plants and have attracted increasing attention due to their many nutraceutical benefits in humans. Fructans, heterogeneous fructose polymers that serve as storage carbohydrates in various plants, represent one of the most important types of natural polysaccharides. Fructans have various physiological and therapeutic effects, which are beneficial to health, and have the ability to prevent or treat various diseases, allowing their wide use in the food, nutraceutical, and pharmaceutical industries. This article reviews the occurrence, metabolism, preparation, characterisation, analysis, and bioactivity of fructans. Further, their molecular weight, monosaccharide composition, linkages, and structural determination are described. Taken together, this review provides a theoretical foundation for further research into the structure–function relationships of fructans, as well as valuable new information and directions for further research and application of fructans in functional foods.

## 1. Introduction

Finding appropriate methods to promote health and good nutrition has been challenging in recent decades and this situation is expected to continue in the immediate future. Recently, product development in the nutritional space has been directed towards functional foods and their ingredients, due to a tremendous increase in demand for healthier foods. Consumers have developed a greater understanding of nutrition and a desire to control their own health, particularly their gastrointestinal health; hence, there has been increased interest in food components that are beneficial to human health, including dietary fibre in the form of polysaccharides and oligosaccharides.

Fructans are non-digestible carbohydrates found in many higher plants as storage polysaccharides and mainly comprise fructose residues with a terminal glucose residue [1]. Fructans are present in many foods and, hence, form part of the typical daily diet. Moreover, since ancient times, fructan-enriched plants have been used as food, animal feed, and folk medicine. Among them, garlic and onion are widely used due to their medicinal properties. Fructans are not only considered as food or food ingredients, but they also offer many benefits to human health, having numerous biological activities, including antioxidant, immunomodulatory, anti-inflammatory, anticancer, antihyperglycemic, and prebiotic activities [2,3]. These functions account for their use in pharmaceutical practice and make them ideal ingredients for application in baked foods, beverages, dairy products, and food preservatives. Nevertheless, research on fructans—especially with the development of biological science tools—has only become sustainable in recent times, and over the past decade, they have attracted increasing attention due to their unique physical, chemical, biochemical, and technological properties.

This review discusses the occurrence, metabolism, preparation, characterisation, analysis, and bioactivity of fructans. Moreover, their molecular weight (MW), monosaccharide composition, linkages, and structural determination are surveyed. This review could provide useful insights and future research directions on fructans for their possible applications.

## 2. Occurrence and Chemistry in Plants

**Figure 1:**
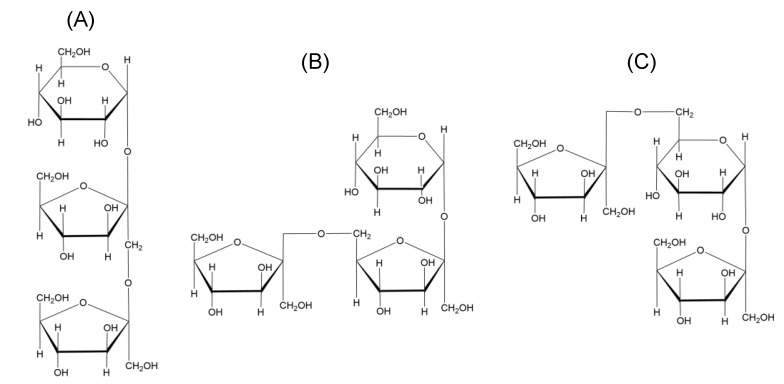
Different types of fructans from higher plants according to the classification of (**A**) 1-kestose; (**B**) 6-kestose; (**C**) neokestose.

Fructans are present in approximately 15% of flowering plants as a major carbohydrate polymer stored in stems and subterranean tissues [4], and occur in a large number of food plants, including wheat, chicory, onion, garlic, and asparagus. The differences in their structures arise from the site of fructose addition, leading to the formation of either 1-kestose, 6-kestose, or 6G-kestose [5]. There are five major classes of fructans in plants (Table 1) on the basis of trisaccharides (Figure 1): linear inulins, inulin neoseries, linear levans, mixed levans (graminans), and levan neoseries.

Structurally, fructans comprising linear glucosyl-α(1→2)-(fructosyl)_n_-β(2→1) polymers with a degree of polymerization (DP) of 3–60 are generally designated as inulins. Plant inulins are commonly linear, with an average DP of 6–12, although chicory inulins have been found to have a small degree of branching (1–2%) [15]. Levans are largely found in bacteria, though some are also found in higher plants. Levans of higher plants are mostly characterized by β(6→2)-fructosyl-fructose linkages, with multiple branches in the form of β(2→1) linkages, while graminans contain both β(2→6)- and β(2→1)-fructosyl-fructose linkages. Muir et al. [16] reported the concentrations of fructans in common foodstuffs, showing that shallots, Jerusalem artichoke, and garlic are rich sources of fructans, with contents of approximately 8.9, 12.2, and 17.4 g/100 g, respectively. Although wheat contains lower levels of fructans (1–4 g/100 g), it is the main contributor of fructans in diet in the UK [17]. Further, although the contents of garlic and onion are low in diet, they contribute a high amount of fructans in diet as they have high fructan contents. However, when considering fructan intake, it is also important to consider specific structural characteristics, including linear or branched structures, linkage types, and chain lengths.

## 3. Metabolism in Plants

In the biosynthesis of fructans in higher plants, sucrose can serve as a fructose donor in the formation of β(2→1) or β(2→6) linkages. Fructan: fructan 1-fructosyltransferase (1-FFT) can synthesize fructan polymers by transferring terminal fructosyl units [18], and, along with sucrose: sucrose-1-fructosyltransferase and sucrose: fructan-6-fructosyltransferase, it is a crucial enzyme for fructan biosynthesis. The identification, functional characterisation, and mapping of fructosyltransferase genes have been reported [19].

Enzymatic degradation of inulin relies on an endo-inulinase (EC 3.2.1.7) that produces fructooligosaccharides with a DP of 2–7. Breakdown of fructans in plants is catalyzed by fructan exohydrolase (FEH), which converts fructans to monosaccharides 

Guangdong Provincial Key Laboratory of Aquatic Product Processing and Safety, Guangdong Province Engineering Laboratory for Marine Biological Products, Guangdong Provincial Engineering Technology Research Center of Seafood, Guangdong Provithat can be used in energy metabolism [20]. A number of different FEH enzymes, including 6-FEH, 6-kestose exohydrolase, and 6G&1-FEH, have been purified from different flowering plants [21,22]. Efforts have been made to understand the molecular aspects of 1-FEH expression, and cDNAs of 1-FEH from grasses and wheat have been used for heterologous expression in yeast [23]. Studies of both biosynthetic and degradative enzymes may provide a foundation for increasing fructan yields in plants.

Fructan contents in plants seem to be positively correlated with drought and cold tolerance, and changes in fructan levels and enzyme activity during cold treatment have been reported [24].

Even when plants initially contain high levels of fructans, reduction in content and deterioration in quality can take place gradually during storage. It has been suggested that fructooligosaccharide levels and variation in FEH activity should be assessed [18]. Degrading enzymes have been shown to signal release from dormancy in onion bulbs, and the effects of cold stress and defoliation on fructan-metabolizing enzymes have been described in *Vernonia herbacea* [25].

High 1-FFT activity together with low FEH activity may prevent depolymerization of fructans to low-DP products [26]. Understanding the expression of 1-FFT and FEH and inhibiting FEH activity using either antisense technology or co-suppression may not only increase fructan contents in plants, but also increase their resistance to drought or frost and ensure higher levels of fructans in diets globally. With advances in transgene technology, transfer of high-performance genes for synthetic enzymes to enhance fructan content and quality in crops is likely to be a focus of research in the next decade.

## 4. Extraction and Purification of Fructans

### 4.1. Extraction

Appropriate sample preparation for fructans requires an understanding of their physical and chemical properties. Fructans are generally water-soluble and can be easily extracted in hot water; for example, fructans from artichoke waste could be extracted in water at 70 °C over 40 min [27]. Fu et al. [28] optimized the extraction of fructans from *Codonopsis pilosula* roots using response surface methodology. Optimized conditions included an extraction temperature of 100 °C, a water/solid ratio of 40 mL/g, and heating for 2.5 h, and gave a fructan yield of 20.2%. Thermal stability of fructans is an important factor that needs to be considered, as fructans may degrade at high temperatures, and caramelization is possible [29]. 

Green extraction approaches for isolation of fructans from natural sources have attracted attention recently because of their high yields, time savings, lower energy requirements, and good performance. New approaches for fructan isolation include ultrasound-, microwave-, and enzyme-assisted extraction, all of which are aimed at accelerating extraction. Compared with thermal extraction, ultrasound- and microwave-assisted extraction are more efficient and time-saving in extracting fructans from *Inula helenium* roots [30]. Shalini et al. [31] used enzyme-assisted extraction to obtain fructans from garlic, using cellulase and hemicellulase in buffer with pH 4.5 at 40 °C and demonstrated that enzyme-assisted extraction gave a higher yield from garlic powder than conventional water extraction.

Fructans are generally precipitated from the initial extract using organic solvents (methanol, ethanol, or acetone) at concentrations of 10–80% (*v*/*v*). The initial product is a complex mixture of fructans and some undesirable compounds, such as proteins. The Sevage method or proteases can be used to remove the proteins [2]. The goal of sample preparation is to remove impurities and increase fructan concentrations prior to purification and analysis.

### 4.2. Purification

**Figure 2:**
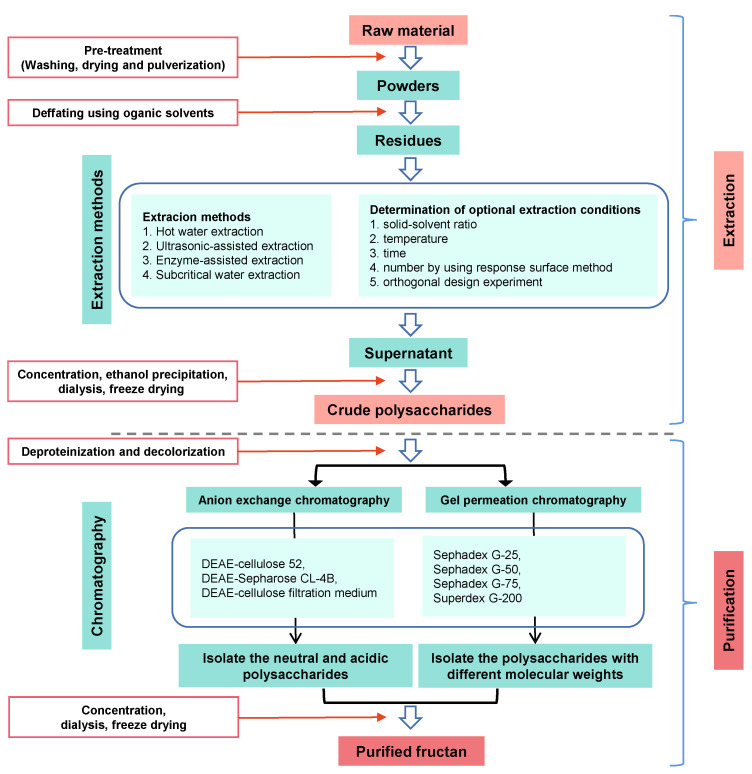
Schematic diagram of extraction and purification of fructans.

The low purity of fructans or the broad MW distribution of fructans has limited the conduction of structure–function relationship studies. Therefore, purification methods that ensure high-purity fructans are necessary. A scheme for isolation, fractionation, purification, and structure determination of fructans is given in Figure 2. The overall process involves extraction of natural fructans from plants, followed by purification using membrane separation and column chromatography. 

Fructans have a high MW due to the multiple fructose units forming polymers with a linear or branched-chain structure. A dialysis membrane or ultrafiltration can be employed to remove low-MW substances [32]. Luiz-Santos et al. [33] used polymeric membranes with different MW cut-offs at a pilot industrial scale for fractionation of agave fructans. Ultrafiltration membranes are also useful for concentrating dilute product streams [34].

The primary purification methods for fructans are based on column chromatography, including ion-exchange, size-exclusion, and activated charcoal chromatography, with varying eluents. Fructans can be purified by size-exclusion chromatography according to MW. For example, a 2.3 kDa fructan from *Aspidopterys obcordata* was purified on a Sephadex LH-20 column (Amersham Pharmacia Biotech AB, Uppsala, Sweden) and eluted with water [35]. *Erwinia herbicola* levan with a MW of approximately 1.4 kDa was purified using a Superose 6 Increase 10/300 GL column and eluted with water [36]. As fructans are neutral polysaccharides, elution in water is also typical in ion-exchange chromatography. A fructan from *Platycodon grandifloras* was subjected to diethylaminoethyl resin ion-exchange chromatography, and a distilled water fraction was collected [37]. Fructan or fructooligosaccharide purification can also be performed using activated charcoal, with elution by distilled water, obtaining fractions with a purity of up to 92% [31].

Recently, preparative hydrophilic interaction liquid chromatography (HILIC) has been used in the purification of fructans. The advantages of HILIC include good retention of polar compounds, high selectivity, and compatibility with several kinds of detectors. Zhang et al. [38] successfully purified 16 fructooligosaccharides from *Atractylodes lancea* using preparative HILIC, demonstrating high purity and high recovery. 

## 5. Characterisation

### 5.1. MW

Numerous studies demonstrated the MW as an important structural feature from the perspective of structure–function relationships. As shown in Table 2, the extraction procedures, structural characteristic, and the biological activity of fructans. Several techniques have been applied for the determination of fructan MW, including high-performance size-exclusion chromatography (HPSEC), laser light scattering, and mass spectrometry (MS). The HPSEC technique assesses the size of fructans based on separation in a gel or resin matrix. Polysaccharides of known MW are used as standards. In one example, standard compounds of MW 70, 40, and 6 kDa, along with maltotriose, sucrose, and glucose were separated on a TSKgel G3000PWXL column (Tosoh, Tokyo, Japan) and used to calculate the MW of three fructan fractions, obtaining equivalent sizes of 4.8, 8.4, and 9.0 sugar units, respectively [9].

One drawback of using size-exclusion chromatography to determine fructan MW is the lack of suitable standards. HPSEC has, thus, been used with a multi-angle laser-light scattering (MALLS, Wyatt Technology, Santa Barbara, CA, USA) detector to assist in determining MW. MALLS is a powerful tool to investigate macromolecular properties and chain conformations. Using MALLS, the MW and root mean square radius of a fructan from *Anemarrhena asphodeloides* Bunge were determined as 2.72 × 10^3^ g/mol and 4 nm, respectively [39]. Similarly, the MW and radius of gyration (Rg) of a fructan from *Bacillus* sp. SCU-E108 were determined to be 3.578 × 10^7^ g/mol and 59.3 nm, respectively. The slope of the correlation between MW and Rg was 0.3, being related to the chain conformation of fructans having a roughly spherical shape in aqueous solution [40].

MS analysis can allow rapid determination of MW for fructans. Sun et al. [35] determined the MW of *A. obcordata* vine fructan by ion trap-time of flight (TOF) MS and observed consecutive losses of fructose (mass-to-charge ratio (*m*/*z*) 162) between two glucosyl moieties, with *m*/*z* 1490.3215 taken to represent [GF8 + NH_4_]^+^. Three fructan fractions isolated from *C. pilosula* roots had DP values of 16, 22, and 31 as determined by matrix-assisted laser desorption ionization (MALDI)-TOF-MS [41]. MALDI-TOF MS appears to be a useful tool for rapid and accurate determination of fructan MW.

**Table 2:** Extraction procedures, structural characteristic, and the biological activity of fructans.

Source	Extraction Procedure	Structural Characteristic	Biological Activity	Ref.
Agave		Mixture of β(2→1) and β(2→6)-Fru*f*	Antioxidant	[42]
*Agave tequilana*	Commercial product	Mixture of β(2→1) and β(2→6)-Fru*f*, DP 7-45	Antiinflammation	[43]
*Anemarrhena asphodeloides*	Hot water (80 °C) extraction	Backbone (2→6)-linked β-D-Fru*f*, MW 2.72 kDa	Neuroprotective and immunoregulatory	[39]
*Atractylodes chinensis*	Enzymatic auxiliary-ultrasonic extraction	Backbone (2→1)-linked β-D-Fru*f*, MW 11.2 kDa	Antitumor	[44]
*Atractylodes macrocephala*	Hot water (80 °C) extraction	α-D-Glcp-(1→(2-β-D-Fru*f*-1)_7_	Anti-weightlessness bone loss	[45]
*Atractylodis macrocephalae*	0.2 mol/L NaOH (100 °C) extraction	Backbone (2→1)-linked β-D-Fru*f*, MW 3.438 kDa	Immunoregulatory	[46]
Asparagus	Hot water (80 °C) extraction	Backbone (2→1)-linked β-D-Fru*f*, DP > 25	Prebiotic	[47]
*Asparagus cochinchinensis*	Hot water extraction	Backbone (2→1)-linked β-D-Fru*f*, MW 2.69 kDa	Regulates gut microbiota	[48]
*Codonopsis pilosula*	Hot water (85 °C) extraction	Backbone (2→1)-linked β-D-Fru*f*, MW 3.6 kDa	Anti-gastric ulcer	[49]
*Codonopsis pilosula*	Ultrasonic extraction (90 °C)	Backbone (2→1)-linked β-D-Fru*f*, DP 16-31	Prebiotic	[41]
*Codonopsis tangshen*	Hot water extraction	Backbone (2→1)-linked β-D-Fru*f*, MW 3.95 kD	Antioxidant and prebiotic	[50]
Jerusalem artichoke	Hot water (70 °C) extraction	Backbone (2→1)-linked β-D-Fru*f*, MW 2.6 kD	Antitumor	[51]
*Lobelia chinensis*	Hot water extraction	α-D-Glc*p*-(1→(1-β-D-Fru*f*-2)_15_ linkage, MW 2.6 kDa	Antiobesity	[52]
*Platycodon grandiflorum*	Hot water (90 °C) extraction	Backbone (2→1)-linked β-D-Fru*f*, MW 12.1 kDa	Regulates gut microbiota	[53]
*Platycodon grandiflorus*	Hot water extraction	Backbone (2→1)-linked β-D-Fru*f*, DP 2–7	Prebiotic and immunoregulatory	[37]

### 5.2. Monosaccharide Composition

The types and molar ratios of monosaccharides in fructans are usually determined using thin-layer chromatography (TLC), paper chromatography (PC), high-performance liquid chromatography (HPLC), gas chromatography (GC), or capillary electrophoresis (CE) [54]. Before analysis, fructans need to undergo acid hydrolysis (using HCl or trifluoroacetic acid (TFA)) or enzymatic hydrolysis to monosaccharides. The strong acid H_2_SO_4_ is seldom used in fructan hydrolysis due to fructose instability in its presence, and the production of heterogeneous hydrolyzed products makes analysis difficult.

TLC and PC are simple methods but have low sensitivity and accuracy in detecting monosaccharides. TLC can be used to separate carbohydrates using an organic mobile phase followed by staining; fructose and glucose can easily be distinguished as differently colored spots on the plate [55]. HPLC has been used extensively to identify constituent monosaccharides and determine their molar ratios. As monosaccharides lack suitable chromophores for absorbance detection, refractive index detectors or evaporative light-scattering detectors (ELSD) are normally used. Sun et al. [48] used HPLC–ELSD to analyze monosaccharides in a fructan from *Asparagus cochinchinensis* that was found to be primarily composed of fructose (93.3%), with a low glucose content (6.7%). HPLC can easily be coupled with MS to analyze monosaccharide composition. In electrospray ionization-MS, sugars are detected in the positive ion mode as sodium adducts ([M + Na]^+^) and fragmentation patterns can be determined [56]. Ions with *m*/*z* 203 correspond to glucose or fructose (sugar 180 + sodium 23), while sucrose ions have *m*/*z* 365, due to loss of one molecule of H_2_O during the formation of the glycosidic link. Similarly, ions with *m*/*z* 527 correspond to kestose. Fructan fragment ions of varying DP can be observed in MS due to glycosidic and cross-ring cleavages.

In determining monosaccharide composition by GC, preparation of chemical derivatives is essential as fructans and monosaccharides have low volatility and stability [57]. In general, one-step derivatization reactions are preferred, although two-step reactions can give better chromatographic outcomes; for example, trimethylsilyl oximes can be obtained using oximation and silylation steps. Derivatization of carbohydrates for GC analyses has been reviewed [58]. GC commonly analyses monosaccharides using FID or MS detectors. Zhang et al. [52] determined the monosaccharide composition of a fructan isolated from *Lobelia chinensis* using hydrolysis and subsequent reduction and acetylation to obtain alditol acetates for GC–MS.

### 5.3. Quantification

The market for fructans has expanded substantially in recent years with increased public awareness of their health benefits. As a result, there has been increasing research interest in determining the quality and quantity of fructans.

Quantification of fructans is challenging, given that plants contain fructans with a DP range of 2–60. Various techniques have been used to quantify fructans, including diverse chromatographic and spectrometric techniques. HPLC is commonly used for reasons of speed, resolution, reproducibility, efficiency, and ease of sample preparation. RID or ELSD may be used for detection but suffers from low sensitivity. HPLC is not suitable for analysis of high-DP fructans, as fructans with DP > 5 give a broad peak. High pressure anion-exchange chromatography with pulsed amperometric detection is suitable for analysis of high-DP fructans, as it can resolve monosaccharides and high-DP fructans at up to a DP of 80 [59]. 

Another reliable method for quantification of fructans involves enzymatic degradation or acid hydrolysis to release glucose and fructose, which are subsequently separated and quantified using GC or HPLC. This approach determines the total content of fructans, but not the DP. The Association of Official Analytical Chemists (AOAC) has developed methods AOAC 2011.25 and AOAC 991.43 that utilize commercial highly purified and specific enzyme kits for hydrolysis of fructans. Glucose and fructose are determined chromatographically before and after hydrolysis [60], and the fructan content is calculated by subtracting the initial glucose, fructose, and sucrose contents from the corresponding final contents. There are some limitations in the use of these AOAC methods to measure total fructans however, as they have low sensitivity and require analytical processes for fructose and glucose.

### 5.4. Linkage Analysis

Linkage analysis of fructans generally relies on methylation-based analysis. This approach is widely used for structural characterisation of fructans and involves methylation of free hydroxyl groups in the intact fructan followed by cleavage of glycosidic bonds using TFA. The hydrolysate containing methylated monosaccharides is chemically reduced using NaBH_4_ or KH to form alditols. Alditol acetates are then prepared and analyzed by GC–MS. From the position of the non-methylated hydroxyl groups, the location of glycosidic bonds in the original fructan can be determined.

Zhang et al. [61] reported the structure of a fructan from *Polygonatum cyrtonema* using a methylation-based approach and showed that this fructan mainly contained (2→1)-linked fructofuranose units. Methylation-based analysis showed a linear inulin extracted from *C. pilosula* to have a backbone mainly comprising (2→1)-linked fructofuranose units without branching [2]. Similarly, a fructan purified from *Artemisia japonica* was found to have a primarily (2→1)-linked fructose backbone with a small content of terminal glucose residues [62].

GC–MS can only determinate the linkage form (n→m), where n gives the numbering of the anomeric carbon and m gives the numbering for the oxygen to which the glycosidic bond is made; α- and β-type anomeric configurations cannot be determined by methylation analysis and require data from nuclear magnetic resonance (NMR) spectroscopy.

### 5.5. Structural Analysis

NMR spectroscopy is a powerful tool for structural characterisation of fructans. NMR is capable of elucidating the primary and secondary structures of biomolecules, as well as dynamic processes in solution. Two-dimensional (2D) NMR is now one of the most commonly used methods for determining the structure of carbohydrate chains. Proton chemical shifts in correlated spectroscopy (COSY) and heteronuclear multiple-quantum coherence (HMQC) spectra allow CH_2_ and CH groups to be distinguished, while nuclear Overhauser effect spectroscopy and total correlation spectroscopy (TOCSY) enable determination of the relative positions of protons within a carbohydrate. Heteronuclear multiple bond correlation (HMBC) spectroscopy can be used to determine linkage positions of carbohydrates and understand which groups are linked to each other.

A combination of one-dimensional (1D) NMR and heteronuclear single quantum coherence (HSQC), COSY, and HMBC spectra revealed that an inulin purified from *Atractylodes macrocephala* had a backbone consisting of α-D-glucopyranose-1→(2-β-D-fructofuranose-1)_7_ [45]. The chemical structure of an inulin-type fructan purified from *Platycodon grandiflorum* roots was shown by 1D- and 2D-NMR (including COSY, TOCSY, HSQC, and HMBC) to include a backbone mainly composed of (2→1)-linked β-D-fructofuranose units with a terminal α-D-glucopyranose [53]. A novel fructan from Radix Codonopsis was shown, based on data obtained from HMBC and COSY spectra, to contain α-D-fructofuranosyl-(2→3)-β-D-fructofuranosyl linkages [63].

A clear understanding of the structure of fructans is necessary to investigating the structure–function relationships and is important to designing bioactive fructans with potential for use in medicine or functional foods.

## 6. Bioactivity

As many food plants contain fructans, it is likely that most daily diets worldwide include fructans. Fructans comprise some of the most important components of water-soluble fibre. A daily fibre intake of about 25 g is recommended in most countries [64]. Fructans are known to be beneficial to human health as a prebiotic with antitumor and antioxidant activities [65,66]. Fructans are a form of soluble fibre that cannot be digested by humans, but they may help prevent obesity and diabetes. Fructans probably offer benefits in the human digestive system by increasing the growth of beneficial bacteria, including *Lactobacillus* and *Bifidobacterium* species in the bowel and decreasing the number of pathogenic bacteria [67]. A diet containing fructans was found to lower insulin, cholesterol, triacylglycerols, and phospholipids in the blood [68]. Fructans also mitigate impaired calcium absorption to inhibit bone loss and osteoporosis [69]. All these effects promote human health, and fructans are now thought to represent one of the most promising potential functional food ingredients.

**Figure 3:**
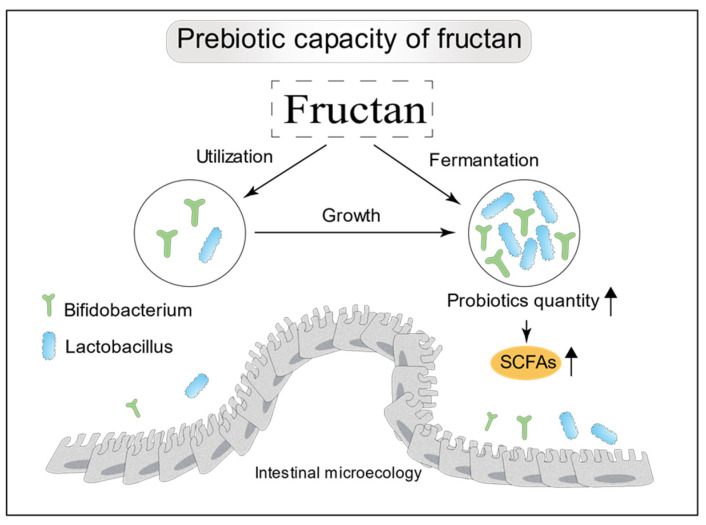
Prebiotic properties of fructans. Principally, they selectively stimulate colonic *Bifidobacterium* and *Lactobacillus*, and increase the production of SCFAs.

The bioactivity of fructans can be expected to vary with structure. Inulin-type fructans typically have a DP of 3–60, while fructooligosaccharides have a DP of 2–20. Inulin and fructooligosaccharides are both fructans with a β(2→1)-type linkage. Bifidobacteria have the ability to intracellularly hydrolyze β(2→1)-D-fructan-fructans, and, thus, can act effectively on these substrates [70], giving them an advantage over other bacteria in the large intestine in terms of utilizing this food source (Figure 3). When inulin-type fructans with a range of DP values were administered to the in vitro Simulator of the Human Intestinal Microbial Ecosystem, it was found that fructans with a higher DP value had stronger prebiotic effects [71]. It is known that oligosaccharides with higher DP values tend to be metabolized more distally in the colon [72]. Another interesting research work has shown that a combination of short- and long-chain fructans has a greater beneficial effect on mineral absorption and modulation of lipid metabolism than does either fraction alone [73]. The prebiotic effectiveness of inulin-type fructans depends not only on DP but also on the dietary dosage. Bouhnik [74] observed correlations between faecal bifidobacteria counts and the dose of fructooligosaccharides fed. Fructans can increase the production of microbiota-derived functional metabolites, such as short-chain fatty acids (SCFAs) (Figure 3). SCFAs are recognized to play an important role in the regulation of appetite and energy intake [75].

While cholesterol and triacylglycerols are normal constituents of human tissues, excessive dietary intake of these substances may increase the risk of body-fat-related diseases, such as cardiovascular diseases. Fructans have been shown to positively modulate digestion and/or metabolism of triacylglycerols. When the dietary intake of fructans increases, blood triacylglycerol levels decrease, both in humans and animals [76]. The liver plays a key role in homeostasis of triacylglycerol-rich lipoproteins, as it can assemble and secrete very-low-density lipoprotein (VLDL). Hepatic output of VLDL can induce lipogenic activity. The hypotriglyceridaemic effect of fructans in vivo may involve suppression of the expression of genes encoding lipogenic enzymes [77].

Oxidative stress arises as a result of loss of antioxidant homeostasis and is associated with the development of various chronic diseases, including coronary heart diseases, neurodegenerative diseases, lung diseases, and cancers [78,79,80]. Fructan from the roots of *Arctium lappa* showed good hydroxyl radical-scavenging and ferrous ion-chelating activities in vitro, while also increasing antioxidant enzyme activity and decreasing the level of malondialdehyde in vivo [81]. A fructan from *A. lappa* also showed significant antioxidant activity in a hydrogen-peroxide-induced HepG2 cell model and a metronidazole-induced zebrafish model [82]. Agave fructans significantly reduced the levels of oxidative stress markers, including thiobarbituric acid-reactive species and carbonyl groups in the brains of overweight mice [42].

In the past several decades, numerous natural polysaccharides have been shown to have significant anticancer activity, as revealed by in vitro, in vivo, and clinical studies [83]. Orally administered inulin can increase the antitumor efficacy of anti-programmed cell death protein-1 immune-checkpoint-blocking therapy. This is because inulin significantly alters the gut microbiota, in particular, by increasing the relative abundance of *Akkermansia*, *Lactobacillus*, and *Roseburia* in the colon and increasing the production of SCFAs. These metabolites can enhance the differentiation of CD8+ T cells into stem-like memory CD8+ T cells that play important roles in tumor immunity [84]. SCFA levels also correlate strongly with inhibition of tumorigenesis and tumor differentiation and with cell cycle arrest and apoptosis, and there is evidence that fructans can alleviate immune stress after radiotherapy [46,85]. 

In short, current pharmaceutical evidence both in vitro and in vivo shows that fructans have good biological activities, especially prebiotic activity. Taking into account the current modern lifestyles of individuals, the amount of dietary fibre consumed by people is lower than the recommended levels; therefore, the application of fructans in food ingredients is an ideal approach to promote human health by maintaining gut health. In addition, fructans have potential application in medicine for the prevention and treatment of cancers, diabetes, and chronic diseases and in the development of medical formulas for patients with organ failure. Since the functional nutritional properties of fructans first became apparent, many studies have been carried out, and it is not possible to cover all of them here. Further, the metabolism of fructans and the mechanisms of their benefits to human health are still not fully clear. More research will be needed, particularly in the form of clinical trials, to further our understanding.

## 7. Future Directions

Further fructan studies will need to integrate various fields, including material, food, agricultural, chemical, analytical, and instrumental science, as well as medicine itself, for the exchange of ideas and technologies. Development of new methodologies will also allow further investigations supporting fructan quantification and structural analysis.

Fructans have become a well-known functional food ingredient, attracting attention from both industry and academic researchers. More in vivo and in vitro data are likely to give a better understanding of the mechanism of action of these saccharides in supporting human health. While the structure of fructans is complicated and there is limited knowledge of structure–function relationships, progress in this area is expected. Future research on fructans should focus on the functions of fructans with different structures, improve the quality and quantity of fructan preparations, and develop appropriate standards to be applied in future research programs.

## Figures and Tables

**Table 1:** Five types of fructans in higher plants.

Type of Fructan	Linear Bond	Formula	Source	Type of Kestose
Linear inulin	β(2→1)	G_1–2_F_1–2_F_n_	Jerusalem artichoke [6], chicory [7]	1-kestose
Inulin neoseries	β(2→1)	mF_2–1_F_2–6_G_1-2_F_1–2_F_n_	Onion [8], asparagus [9], agave [10]	6G-kestose
Linear levan	β(2→6)	G_1–2_F_6–2_F_n_	Rye grass [11]	6-kestose
Mixed levan	β(2→1) β(2→6)	G_1–2_F_1 (6–2)_F m_–2_F	Wheat [12], rye, barley [13]	1-kestose 6-kestose
Levan neoseries	β(2→1) β(2→6)	F_2(6–2)_Fm_–6_G_1–2_F_1 (6–2)_F _n–2_F	Oat [14]	6G-kestose
